# Protein cross‐linking and the Maillard reaction decrease the solubility of milk protein concentrates

**DOI:** 10.1002/fsn3.657

**Published:** 2018-05-02

**Authors:** Fengjiao Fan, Meng Liu, Pujie Shi, Xianbing Xu, Weihong Lu, Zhenyu Wang, Ming Du

**Affiliations:** ^1^ School of Food Science and Technology National Engineering Research Center of Seafood Dalian Polytechnic University Dalian China; ^2^ Department of Food Science and Engineering Harbin Institute of Technology Harbin China

**Keywords:** Maillard reaction, mechanism, milk protein concentrate, protein cross‐linking, solubility

## Abstract

Milk protein concentrate (MPC) is a widely used material in the food industry. However, despite its widespread use, the mechanism underlying the decreased solubility of MPC that occurs during storage has not yet been clarified. In this study, the solubility changes, protein cross‐linking, and Maillard reaction and the relationships between them were investigated in modified MPC powders (MMPC) containing different concentrations of protein and/or lactose stored at 50°C for 15–45 days. The results demonstrated that both the protein and lactose contents affected solubility. The proteins interacted through hydrogen bonding, disulfide bonding, hydrophobic interactions, and nondisulphide covalent bonding, which led to cross‐linking. The Maillard reaction promoted protein cross‐linking and was in turn influenced by protein cross‐linking. The Maillard reaction was slower when the degree of protein cross‐linking was greater. These results improve our understanding of the mechanism leading to poor solubility of MPC powders during storage.

## 1 INTRODUCTION

Milk protein concentrate (MPC) is a powder manufactured from skim milk through membrane separation and spray drying. The protein content of MPC powders ranges from 40 to 90%, based on total solid content (Kelly, 2011; Sikand, Tong, Roy, Rodriguez‐Saona, & Murray, 2011). MPCs are named according to their protein content. For example, MPC85 contains approximately 85% protein content. MPC powders are widely applied in cheese, yogurt, beverage, and confection manufacturing due to their desirable functional properties and nutritional qualities (Farkye & Yim, 2003; Francolino, Locci, Ghiglietti, Iezzi, & Mucchetti, 2010). The solubility of specific MPC powders is closely related to their various functional properties, such as emulsification, gelation, and foaming (Dybowska, 2008; Sandra & Corredig, 2013; Ye, 2011). Despite their widespread use, the solubility of MPC powders gradually decreases during storage, which limits their application (De Castro‐Morel & Harper, 2002; (Anema, Pinder, Hunter, & Hemar, 2006; Havea, 2006). For example, it was reported that the solubility of MPC85 was 53% and 32% after 2 days and 24 months of storage at 20°C, respectively (Havea, 2006). Thus, various approaches have been used to improve the solubility of MPC powders, such as raising the water temperature and extending hydration time (Fang, Selomulya, Ainsworth, Palmer, & Chen, 2011; Kuo & Harper, 2003). However, after extended storage, the solubility was still low, even at a high dissolution water temperature (McKenna, 2000). Generally, it is believed that the loss of solubility is linked to processing. Some studies have suggested that ultrasonication and the addition of salt could increase the solubility (Mao, Tong, Gualco, & Vink, 2012; McCarthy, Kelly, Maher, & Fenelon, 2014; Sikand, Tong, & Walker, 2013). For example, the solubility of stored MPC80 was increased from 63 to 100% by the addition of 50–150 mM NaCl during the diafiltration step of manufacturing. However, these studies did not fundamentally solve the low solubility of stored MPC. Thus, determination of the mechanism underlying the decreased solubility of MPC powders during storage is very important for improving its functionality and expanding its applications in the food industry.

Protein cross‐linking, the Maillard reaction, lipid oxidation, lipolysis, and proteolysis of MPC powders may occur during storage (Gaiani et al., 2007; Le, Holland, Bhandari, Alewood, & Deeth, 2013). Protein cross‐linking and the Maillard reaction are the two main factors that contribute to the decreased solubility of MPC powders. The objective of this study was to investigate the changes in the chemical and physical properties of modified commercial MPC powders after storage for a certain time period at 50°C. The solubility, protein cross‐linking, and Maillard reaction were examined in stored MMPC powders to clarify the mechanism underlying the solubility decrease.

## 2 MATERIALS AND METHODS

### 2.1 Preparation of MMPC samples

Fresh MPC85 powder (MPC containing 85% protein) was purchased from Idaho Milk Products (Jerome, ID, USA). Fresh MPC70 powder (MPC containing 70% protein) was purchased from Dairy Farmers of America (Kansas City, KS, USA). MPC85 and MPC70 powders with no sugar (MPC85‐NS and MPC70‐NS, respectively) were obtained by dialyzing out the lactose and then freeze‐drying. Using Kjeldahl Method and phenol‐sulfuric acid method, the final protein and sugar contents of MPC85‐NS were 92.76% and 0.1%, respectively, and the protein and sugar contents of MPC70‐NS were 89.32% and 0.1%, respectively (Table 1). Modified MPC powders with different amounts of protein (MMPC‐P) were obtained by mixing MPC85‐NS or MPC70‐NS with sucrose, and the measured protein content of modified MPC85 (MMPC85), modified MPC70 (MMPC70), modified MPC55 (MMPC55), and modified MPC40 (MMPC40) was 85%, 70%, 55%, and 40%, respectively (Table 2). Modified MPC powders with different amounts of lactose (MMPC‐L) were prepared from MPC70 through hydration, dialysis for various lengths of time, and vacuum freeze‐drying. The measured protein content of the MMPC‐L samples modified MPC75 (MMPC75), modified MPC77 (MMPC77), modified MPC78 (MMPC78), and modified MPC80 (MMPC80) was 75%, 77%, 78%, and 80%, respectively, and the measured lactose content was 14%, 7%, 1.4%, and 0.14%, respectively (Table 3). All powders were stored at 50 ± 1°C for up to 45 days. The control powders were stored at −20°C. The solubility, electrophoretic pattern, and color of each MMPC powder were examined after 15, 30, and 45 days of storage.

**Table 1:** Protein and sugar content of MPC‐NS

Samples	MPC85‐NS	MPC70‐NS
Protein (%)	92.76 ± 0.48^a^	89.32 ± 1.78^a^
Sugar (%)	0.10 ± 0.02	0.10 ± 0.01

MPC‐NS, MPC powders with no sugars; MPC powders with no sugar (MPC85‐NS and MPC70‐NS) were obtained by dialyzing total lactose and vacuum freeze‐drying.

a The final protein contents of MPC85‐NS and MPC70‐NS were determined; All values are the mean ± SD (*n *= 3).

**Table 2:** Preparation of MMPC powders with different protein levels (no reducing sugar)

Samples	MMPC85	MMPC70	MMPC55	MMPC40
MPC85‐NS (g)	18.30	—	—	—
MPC70‐NS (g)	—	15.70	12.30	9.00
Sucrose (g)	1.70	4.30	7.70	11.00
Total mass (g)	20.00	20.00	20.00	20.00
Protein content (%)	85.00	70.00	55.00	40.00

MMPC powders with different protein levels (MMPC‐P) were obtained by mixing MPC85‐NS or MPC70‐NS with sucrose as the nonreducing sugar;—represent no added.

**Table 3:** Preparation of MMPC powders with different lactose levels

Samples	MMPC75	MMPC77	MMPC78	MMPC80
Lactose content (%)	14.00	7.00	1.40	0.14
MPC70 (g)	20.00	20.00	20.00	20.00
Dialysis time (h)	0	5	30	48
Protein content (%)^a^	75.25	77.75	78.75	80.25

MMPC powders with different sugar levels (MMPC‐L) were prepared from MPC70 by adjusting dialysis time and produced by vacuum freeze‐drying, and the compositions of these samples are shown.

a Final protein contents of the samples.

### 2.2 Solubility assay

The rehydration procedure was carried out as described previously, with a minor modification (Arnaud Mimouni, Deeth, Whittaker, Gidley, & Bhandari, 2009). MMPC solutions (4%) were reconstituted by stirring the powders in distilled water at 1500 rpm for 10 min at 30°C. The rehydrated solutions were then centrifuged at 2400 × *g* for 10 min at 10°C. The centrifugation sediments and rehydrated solutions were subjected to solid determination by oven‐drying (at 105°C) to a constant weight. The solubility of MMPC powders was calculated using the equation below.

$$\text{Solubility}\left(\%\right)=\frac{\text{Solids in the solution}-\text{Solids in the sediment}}{\text{Solids in the solution}}\times 100\%$$

### 2.3 Polyacrylamide gel electrophoresis

The protein composition of the supernatant was determined using sodium dodecyl sulfate‐polyacrylamide gel electrophoresis (SDS‐PAGE) technique under native (no β‐mercaptoethanol) and reducing (with β‐mercaptoethanol) conditions in a modified Laemmli system (Laemmli, 1970) with 5% acrylamide in the stacking gel and 12% acrylamide in the separating gel. Protein bands were visualized by staining with Coomassie R250 brilliant blue. Standard proteins (Thermo Scientific, Shanghai, China) were used for molecular mass determination as follows: β‐galactosidase (116 kDa), bovine serum albumin (66.2 kDa), ovalbumin (45 kDa), lactate dehydrogenase (35 kDa), REase Bsp981 (25 kDa), β‐lactoglobulin (18.4 kDa), and lysozyme (14.4 kDa). After staining and destaining, the gels were scanned using a Bio‐Rad Gel Doc XR system and analyzed with Quantity One software.

### 2.4 Changes in color

The surface color properties of the MMPC powders were measured using a Color Meter ZE6000 (Nippon Denshoku Industries, Tokyo, Japan), and the results were expressed as L*, a*, and b* values (Morales & Van Boekel, 1998). The color values reported are the means of three measurements.

### 2.5 Statistical analysis

Data were analyzed by analysis of variance and the F test using the Statistical Product and Service Solutions (SPSS) 11.0 software package (SPSS Inc., Chicago, IL, USA). *p* values <0.05 were considered statistically significant.

## 3 RESULTS AND DISCUSSION

### 3.1 Effects of protein content on solubility

**Figure 1:**
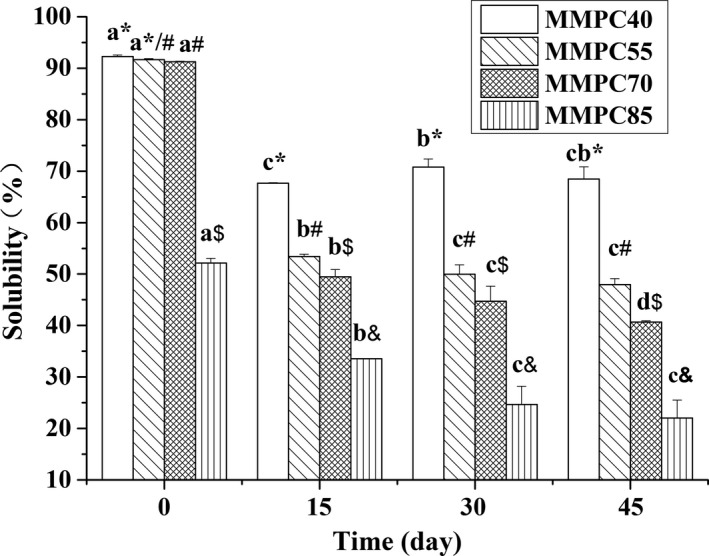
Changes in the solubility of MMPC‐P samples. Error bars indicate the standard deviation. Different letters (a, b, c, and d) indicate significant differences among the same sample (e.g., MMPC40) at different time points. Symbols (*, #, $, and &) indicate significant differences among different samples at the same time point. Different letters and symbols indicate significant differences (*p *<* *0.05)

The solubility of MMPC‐P samples stored at 50°C for up to 45 days is shown in Figure 1. MMPC40, MMPC55, and MMPC70 initially showed relatively good solubility. Then, the solubility decreased rapidly during storage. The MMPC85 sample showed lower solubility than others, even at the beginning of storage (day 0). As shown in Table 2, the MMPC85 was the mixture of MPC85‐NS and sucrose, MMPC70, MMPC55, and MMPC40 were derived by mixing MPC70‐NS and sucrose. It took much longer to rehydrate powders with high protein content; thus, MMPC85 showed lower solubility than MMPC70 under the same conditions. As for the stored MPC powders, the internal structure of the casein micelles remained unchanged, whereas the interface became porous and gel‐like due to the cross‐linking of α‐caseins and β‐caseins (Mata, Udabage, & Gilbert, 2011; Mimouni, Deeth, Whittaker, Gidley, & Bhandari, 2010). The porous gel‐like structure limited the dispersion of micelles into water and contributed to the loss of solubility during storage (Mimouni et al., 2009).

After 15 days, the solubility of all the MMPC‐P samples decreased significantly. The solubility continuously declined during the 30 days, and MMPC70 showed the greatest decline among the four groups. After 45 days, the solubility of MMPC40, MMPC55, MMPC70, and MMPC85 declined to 74.21%, 52.33%, 44.57%, and 42.29%, respectively, which indicates that the solubility of MMPC powders is closely related to the protein content. The possibility of protein–protein interactions increases at higher protein content; therefore, at the same storage time point, the MMPC85 sample had the lowest solubility. Protein unfolding and surface hydrophobicity were also increased when MPC powders were stored at high temperatures, which could lead to protein–protein interactions (Haque, Bhandari, Gidley, Deeth, & Whittaker, 2011; Haque et al., 2010).

### 3.2 Effects of lactose content on solubility

**Figure 2:**
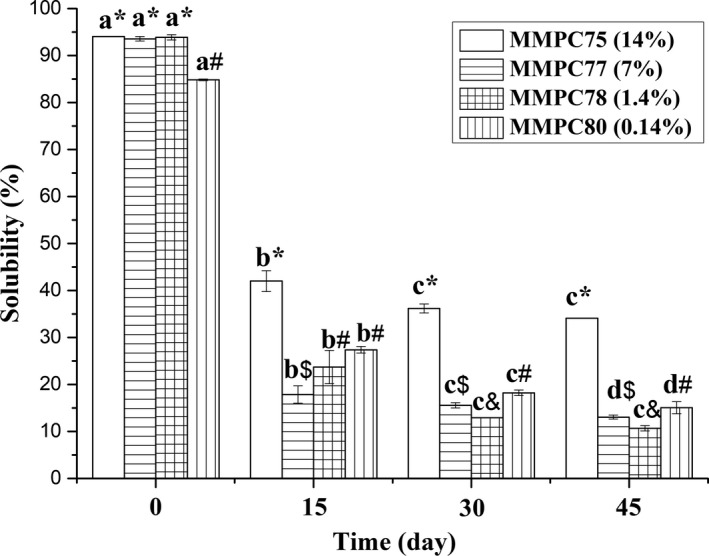
Changes in the solubility of MMPC‐L samples. Error bars indicate standard deviations. Different letters (a, b, c, and d) indicate significant differences between the same samples at different time points. Symbols (*, #, $, and &) indicate significant differences among the different samples at the same time point. Different letters and symbols indicate significant differences (*p *< *0*.05)

The solubility changes of MMPC‐L samples stored at 50°C for various time points are shown in Figure 2. Compared with the MMPC‐P samples, the solubility of the MMPC‐L samples decreased significantly faster. In addition, MMPC75 had the lowest protein concentration, which also contributed to its higher solubility.

After 15 days, the solubility of all MMPC‐L powders decreased sharply, and the solubility of MMPC75 and MMPC80 was higher than the other samples. These results indicated that both the protein and lactose contents influenced the solubility of the powders. Over the next 30 days, the solubility of all the MMPC‐L powders continued to decrease. At 45 days, the solubility of MMPC75, MMPC77, MMPC78, and MMPC80 was reduced to 36.24%, 13.93%, 11.36%, and 17.76% of the initial value, respectively. At this time point, the solubility of MMPC80 was higher than that of MMPC77 and MMPC78. These results suggest that the Maillard reaction may also influence the solubility of MMPC powders. Reconstitution may also influence the solubility of the powders due to protein denaturation. For example, the solubility of fresh powder MMPC75 was higher than that of the reconstituted powders MMPC77, MMPC78, and MMPC80.

We concluded that during storage, the casein in commercial MPC85 powder was lactosylated (Anema et al., 2006). The Maillard reaction can produce glyoxal and methylglyoxal, two substances that further promote protein cross‐linking, which indirectly influence solubility. We detected a correlation between the Maillard reaction and the solubility of commercial MPC80, and we concluded that casein and α‐lactalbumin were involved in the Maillard reaction (Le, Bhandari, & Deeth, 2011; Le, Deeth, Bhandari, Alewood, & Holland, 2012). However, further studies are required to investigate the relationship between protein cross‐linking and the Maillard reaction in modified MPC powders.

### 3.3 SDS‐PAGE analysis

**Figure 3:**
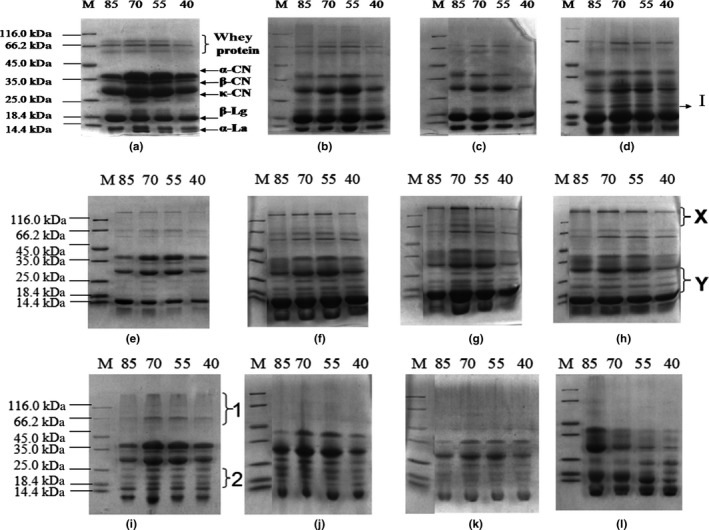
Electrophoretic separation of the proteins in the MMPC‐P samples. M: Marker, 85: MMPC85, 70: MMPC70, 55: MMPC50, 40: MMPC40. (a, b, c and d): SDS‐PAGE (with SDS, with β‐mercaptoethanol) of MMPC‐P samples stored for 0, 15, 30, and 45 days, respectively. (e, f, g and h): PAGE (with SDS, no β‐mercaptoethanol) of MMPC‐P samples stored for 0, 15, 30, and 45 days, respectively. (i, j, k, and l): Native‐PAGE (no SDS, no β‐mercaptoethanol) of MMPC‐P samples stored for 0, 15, 30, and 45 days, respectively

The SDS‐PAGE of MMPC‐P samples after 0, 15, 30, and 45 days of storage is shown in Figure 3. The changes in the MMPC‐P protein pattern increased with storage time. The molecular weights of bands observed in the SDS‐PAGE are summarized in Table 4. There were reductions in the densities of the α‐, β‐, and κ‐casein bands after 15, 30, and 45 days when compared to the densities of those of protein bands on day 0. Cross‐linking between casein and others proteins could account for the insolubility of MPC powders with higher protein content. MMPC85 showed that most obvious changes among the MMPC‐P samples, which was consistent with the changes in the solubility of the MMPC‐P samples.

**Table 4:** The molecular weight of new bands

Name	MMPC‐P (kDa)				MMPC‐L (kDa)			
	0 day	15 days	30 days	45 days	0 day	15 days	30 days	45 days
I	23.24	23.24	23.24	23.24	22.73	22.73	—	22.73
X	200.28	101.57	101.57	200.28	—	94.34	94.34	94.34
		200.28	200.28			136.45	136.45	136.45
						191.61	191.61	191.61
Y	19.80	19.80	19.80	19.80	19.68	19.68	19.68	19.68
	22.39	22.39	22.39	22.39	22.12	22.12	22.12	22.12
	26.31	26.31	26.31	26.31	26.17	26.17	26.17	26.17
1	67.27	—	—	—	70.88	—	—	—
2	18.76	18.76	18.76	18.81	17.03	17.03	17.03	19.19
	21.8	21.8	21.8	22.9	20.33	20.33	20.33	23.32
	25.5	25.5	25.5		24.3	24.3	24.3	

—, without the band.

As the hydrophobic bonds and hydrogen bonding were disrupted by the dissociating agent SDS, and the disulfide bonds were reduced with β‐mercaptoethanol, band I in (d) was generated through nondisulphide covalent bonding. The new bands, named X and Y in (e), (f), (g), and (h), contained disulfide bonds, and there were high molecular weight bands at position X and three clear bands at position Y. The density of the bands at position Y increased with increasing storage time. The number of the bands at position X changed with storage time. At 15 and 30 days, there were two bands at position X in MMPC70 and MMPC55. The native‐PAGE showed that the protein at position 1 gradually aggregated into high molecular weight bands and then became trapped in the well; therefore, there was no protein band at position 1 on days 15–45. The protein at position 2 also formed large protein aggregates through nondisulphide covalent bonding. At 45 days, the density of the bands at position 2 increased, and the number of bands changed from 3 to 2 due to the formation of large protein aggregates through nondisulphide covalent bonding.

**Figure 4:**
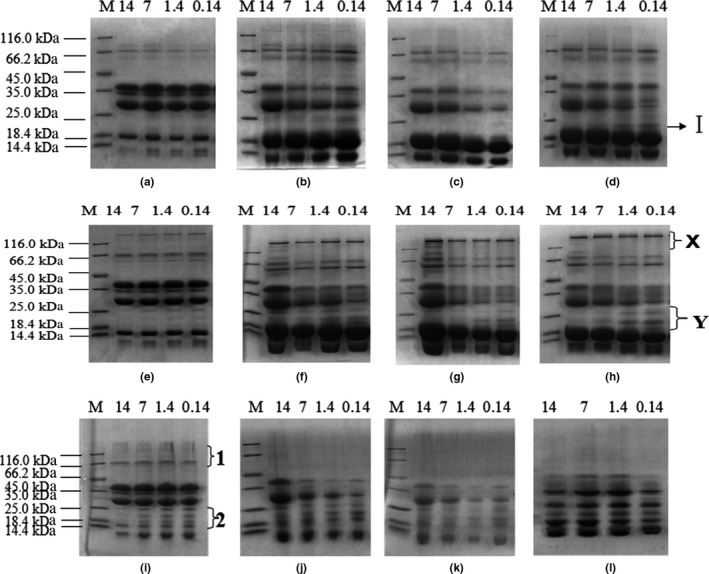
Electrophoresis of MMPC‐L samples. M: Marker, 14: MMPC75, 7: MMPC77, 1.4: MMPC78, 0.14: MMPC80. (a, b, c, and d): SDS‐PAGE (with SDS, with β‐mercaptoethanol) of MMPC‐L samples stored for 0, 15, 30, and 45 days, respectively. (e, f, g, and h): PAGE (with SDS, no β‐mercaptoethanol) of MMPC‐L samples stored for 0, 15, 30, and 45 days, respectively. (i, j, k, l): Native‐PAGE (no SDS, no β‐mercaptoethanol) of MMPC‐L samples stored for 0, 15, 30, and 45 days, respectively

The gels of the MMPC‐L samples after 0, 15, 30, and 45 days are shown in Figure 4. On the whole, the changes in the MMPC‐L samples were greater than those observed for the MMPC‐P samples. The molecular weights of the bands with the most remarkable changes are shown in Table 4. In addition, there were also reductions in the densities of the α‐, β‐, and κ‐casein bands in the MMPC‐L powders during storage. The SDS‐PAGE of MMPC78 (1.4%) and MMPC80 (0.14%) after storage for 45 days also showed band I, which was generated through nondisulphide covalent bonding. These results showed that protein cross‐linking generated this new band.

As shown in Figure 4e–h, there were high molecular weight bands at position X and three clear bands at position Y. The density of the bands at position Y increased, and the number of the bands at position X changed with increasing storage time. At 30 and 45 days, there were three clear bands in MMPC75 (14%) at position X. Thus, lactose content had an effect on protein cross‐linking; high lactose content promoted the formation of high molecular weight proteins, whereas low content of lactose promoted the formation of new low molecular weight proteins. The gel of (i), (j), (k), and (l) in Figure 4 showed that the protein at position 1 gradually aggregated into larger molecules and then became trapped in the well. Therefore, there was no band at position 1 at 15, 30, and 45 days. At 45 days, the density of the bands at position 2 increased and the number of bands changed from 3 to 2 due to the formation of large protein aggregates through nondisulphide covalent bonding.

As shown in Table 4, the molecular weights of the new bands in the MMPC‐L samples were lower than those in the MMPC‐P samples. In addition, at position X, one band (136.45 kDa) was absent in the MMPC‐P powders but was present in MMPC75 (an MMPC‐L sample), which indicated that there were different protein‐crosslinks in the MMPC‐P and MMPC‐L samples, and the 136.45 kDa protein was probably produced via the Maillard reaction. The electrophoresis results revealed that hydrogen bonding, disulfide bonding, hydrophobic interactions, and nondisulphide covalent bonding were involved in the protein cross‐linking of stored MPCs.

### 3.4 Changes in color during storage

**Figure 5:**
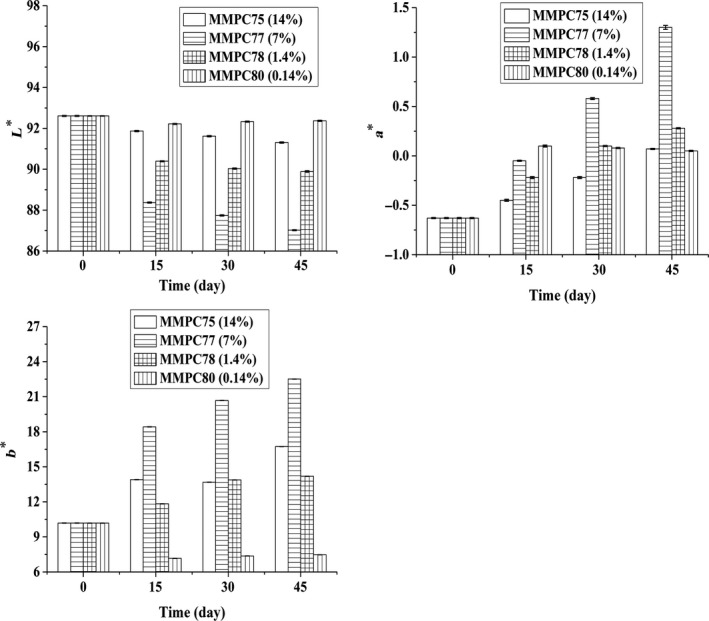
Changes in the color of MMPC‐L samples during storage. L* represents lightness, and a* and b* represent redness and yellowness, respectively

The L*, a*, and b* values were used to assess the changes in color of MMPC‐L samples during storage. These color values, L*, a*, and b*, represent lightness (or whiteness), redness (or greenness), and yellowness (or blueness), respectively. Figure 5 shows the changes in the color of the MMPC‐S samples during storage. The decrease in the L* value represents a decrease in lightness and a corresponding increase in darkness. The color of MMPC77 (7%) was the darkest, and the color of MMPC40 was the lightest. The increases in a* and b* represent increases in redness and yellowness, respectively. MMPC77 (7%) showed significant changes in L*, a*, and b* during storage, whereas the other samples only showed gradual changes. There was significant correspondence between the solubility change in MMPC77 (7%) during storage and the presence of Maillard reaction products, which caused the protein cross‐linking that was responsible for the decreased solubility (Le, Bhandari, Holland, & Deeth, 2011; Le et al., 2013). In the Maillard reaction, lactose binds to protein at lysine residues, resulting in the formation of the first‐stage Maillard reaction product, lactulosyllysine. Moreover, advanced glycation end products can lead to browning and the formation of high molecular weight protein complexes (Mottram, Wedzicha, & Dodson, 2002; Singh, 1991).

Compared with MMPC75 (14%), MMPC77 (7%) showed more remarkable changes in color. Thus, the occurrence of the Maillard reaction in MPC was not linearly proportional to the lactose content. The protein and lactose contents in MMPC77, 77% protein and 7% lactose (a mass ratio of 11:1), facilitated the Maillard reaction.

## 4 CONCLUSIONS

In summary, the solubility of all modified MPC powders decreased during storage, and protein cross‐linking was the major reason for the solubility decrease in MPC powders during storage. The protein molecules interacted through hydrogen bonding, disulfide bonding, hydrophobic interactions, and nondisulphide covalent bonding. In addition, the Maillard reaction also decreased solubility during storage. A small amount of lactose in the MPC reacted with proteins or amino acids with free amino groups; this process formed products that promoted protein cross‐linking, and in turn, the Maillard reaction influenced protein cross‐linking.

## CONFLICT OF INTEREST

The authors declare no conflict of interests.
